# *Morinda citrifolia *and the pharmaceutical industry: technological prospecting and potential

**DOI:** 10.1186/1753-6561-8-S4-P196

**Published:** 2014-10-01

**Authors:** Clauberto Oliveira, Ricardo Albuquerque-Junior, Mairim Serafini, Gabriel Silva, Adriano Araujo

**Affiliations:** 1 Universidade Tiradentes, Aracaju, SE, Brasil; 2 Universidade Federal de Sergipe, SE, Brasil

## Introduction

Medicinal plants have been used since ancient times as medicines for the treatment of a wide range of diseases. Morinda citrifolia L (Noni) has been used in folk remedies by Polynesians for over 2000 years, and is reported to have a broad range of therapeutic effects, including effects against headache, fever, arthritis, gingivitis, respiratory disorders, infections, tuberculosis and diabetes, it is commonly used as herbal medicines and cosmetics.

## Objectives

This study aimed to map the patent applications, analyzing the potential and the evolution of technological capabilities through the translated patent applications with regard to the *Morinda citrifolia*.

## Methods

Prospecting was carried out in the databases of the European Patent Office (Espacenet/EP), World Intellectual Property Organization (WIPO), United States Patent and Trademark Office (USPTO) and the Instituto Nacional de Propriedade Intelectual (INPI) of the Brazil. The keywords used was *Morinda citrifolia*, in addition to the substantives skin, cosmetic, antioxidant and dermatological. The research fields filled with the keywords were "title" and "abstract", and subsequently the research was restricted application the field "international patent classification (IPC)" with the codes A61K (regarding preparations for medical, dental or hygiene purposes) and A61Q (regarding the specific use of cosmetics or similar preparations for personal hygiene).

## Results

Using the keyword Morinda citrifolia, 391 patent applications were found, 9 (2.3%) in the PTO, 310 (79.28%) in the Espacenet, 23 (5.88%) in the USPTO and 49 (12.53 %) in the WIPO. The selection of patents was restricted combining the words skin (total of 28 applications, 24 of them found in the Espacenet, 01 in the INPI, and 03 in the WIPO); cosmetic (15 applications in the Espacenet), antioxidant (total of 05 applications, 05 in the INPI, 01 in the Espacent and 02 in the WIPO); and dermatological (01 application in the Espacent). Japan and United Staes were the main depositor countries. The highest number of patents obtained with the combination of the keywords *Morinda, citrifolia *and IPC A61K presented the subcode A61K36, representing pharmaceutical preparations characterized by active organic. Furthermore, the highest number of patents among codes A61Q laid in the subcode A61Q1, representing preparations for skin cleansing. The largest number of applications occurred between 2002 and 2009.

## Conclusions

The data showed that the area related to technological research with *Morinda citrifolia *is promising, and that since 2000 there has been a growing increase in the number of patents, particularly in the years 2005 and 2007. The United States and Japan lead the ranking of depositor countries. The largest technology is involved in the production of cosmetics for skin care, and most exploited in the makeup production and in medicinal preparations.
